# A Practical Treatise on the Diseases Peculiar to Women

**Published:** 1841-01-01

**Authors:** 


### A PRACTICAL TREATISE ON THE DISEASES PECULIAR TO WOMEN. By *Samuel Ashwell*, M.D. Part 1. FUNCTIONAL DISEASES. London, 1840. Pp. 200. Octavo. Highley.

THIS is an excellent practical work, the result of Dr. Ashwell's extensive experience as obstetric physician at Guy's. A faithful analysis of its contents will enable our readers to judge of their value.

### CHAP. I.—CHLOROSIS.

*Symptoms.*—Dr. Ashwell distinguishes three forms of this disease, viz. a mild and incipient, and inveterate and confirmed, and a complicated form. The *mild* form, often preceded from even early infancy by a greater or less state of debility, only manifests its peculiar nature at the period of puberty, when the non-establishment of the various changes incidental to that epoch excites maternal solicitude, ever awake to all irregularities of the menstrual functions. We need not detail the symptoms which manifest themselves; the pasty complexion, listless movements, capricious appetite, and numberless ailments of the chlorotic girl, are known even to the tyro. When the disease becomes *inveterate* and *confirmed* the sufferings are indeed great, the depression of spirits is distressing, the most extraordinary substances are consumed in place of natural articles of diet; the complexion assumes a dirty green cast, while the lips, tongue, and fauces are bloodless; the digestive organs are materially disordered, whence vomiting, constipation, and sometimes diarrhœa. The head is the seat of innumerable pains and anomalous sensations; the cellular texture of the body becomes more or less infiltrated, and the general surface is cold and pallid; it is sometimes very dry, the hair losing its brightness, and the finger-nails becoming brittle and split. Many symptoms, at this period of the disease, may easily be mistaken for indications of structural disease of some of the great organs of the body.

#### CAUSES OF CHLOROSIS.

" *Predisposing.*—A delicate, feeble, and undeveloped constitution, where the circulation and nervous power are inadequately excited to perfect the organization of the body, in consequence of which the evolution of the ovaries is delayed, and their peculiar influence on the system, and particularly on the uterus, is withheld; thus, puberty is only imperfectly or perhaps not at all established, and menstruation, which must be preceded by puberty, is absent. At a later period of life, when even married women and widows are the subjects of chlorosis, its predisposing cause is most frequently derangement of menstruation; there is either retention, irregularity, or pain and difficulty in the performance of the function. Nor must it be forgotten, that profuse menstruation, menorrhagia, and chronic leucorrhœa, may induce chlorosis." P. 7.

Unhealthy localities, the unnatural habits of the rich, the privations of the poor, and indeed all causes exerting an enfeebling effect upon the system, especially in early life, may predispose to the disease. Dr. Ashwell has seen one or two well-marked cases in men.

*Exciting.*—Circumstances exerting a depressing effect upon the mind, as disappointed affections, &c. &c. Diseases and habits which produce a debilitating effect upon the system in general and the sexual in particular, as amenorrhœa, menorrhagia, excessive venery, manusturpation, &c.

### PATHOLOGY OF CHLOROSIS.

"It may probably be fairly assumed, certainly it is the most prevalent opinion, that chlorosis primarily depends upon a morbid condition of the blood, which secondarily affects the ovaries and uterus, by retarding their growth. This opinion is supported by the fact, that in the blood of chlorotic patients there is an encreased proportion of the serum, with a marked diminution of crassamentum. This has always been my view of the disease; nor would it be difficult to trace to this morbid condition of the blood many, nearly all, the different theories that have been propounded." 8.

### DIAGNOSIS.

Some of the symptoms of chlorosis, as cephalgia, dyspnœa, palpitation, &c., are too often treated by the ignorant or superficial observer as of inflammatory origin. "From the want of this caution, I have witnessed the very injurious consequences of such mistakes, the practitioner having forgotten, what in female diseases it is peculiarly important to remember, that the severity of the pain, and the rapidity of the pulse, are generally indications of irritability and excitement, not of inflammation; demanding narcotics, carminatives, and at the most, counter-irritation, not bleeding, active purging, and spare diet."

### COMPLICATIONS OF CHLOROSIS.

*Complication with Amenorrhœa.*—It is an error frequently committed to suppose chlorosis and amenorrhœa to be convertible terms. There cannot be chlorosis without more or less amenorrhœa, yet amenorrhœa often exists without any degree of chlorosis. The complication of the two may arise, either, as it generally does, from the persistence of chlorosis preventing or arresting menstruation, or, occasionally, from the occurrence of amenorrhœa after healthy menstruation has been established. These complicated cases are those which, if promptly and effectually treated (usually by tonics) so often terminate in health. In some instances, however, the treatment is difficult and protracted, and great alarm is felt lest more serious complications should have occurred: indeed, advice is not sought in these cases, sometimes, until some vital organ has become involved. "I have often, during the last few years, been requested to treat such patients, of whom, had I judged from what I heard, I should not have predicted any danger; and yet, on careful inquiry—and in some instances at first sight—I have been convinced that the case was all but hopeless." Although these protracted cases, under a concurrence of favorable circumstances, sometimes do well, yet, ordinarily, some vital organ becomes the seat of fatal disease, if proper remedies be not resorted to.

*Complication with Hæmatemesis.*—This is by no means uncommon in cases of chlorosis with amenorrhœa. The treatment may have been to a certain degree successful, the quality of the blood may have become improved, and, during the continuance of the amenorrhœa, engorgement of the digestive organs occurring, a vomiting of blood is the result, and it often continues to recur at intervals not very unlike the catamenial periods. Sometimes alarming anæmia results. These are the cases in which local emmenagogues are so useful. The quality of blood has now become sufficiently good, and endeavours must be made to divert it into its natural channels.

*Complication with Derangement of the Digestive Organs.*—These organs are always more or less affected, but sometimes very prominently so. Dr. Ashwell truly observes, that the real matter of astonishment is, that the powers of life are so long maintained, when we consider the nature and insufficient quantity of food taken by the chlorotic patient. Indeed, the vital powers are often reduced to the lowest ebb, and the recovery of the patient pronounced impossible; yet, the author's experience has taught him, that, where no predisposition to phthisis exists, there is no complication of the disease, "which affords such ample scope and reward to judicious, persevering, and observant treatment. It is rare for a structural change to occur in the stomach, liver, or intestines, in the most protracted form of the disease, although it is common to see the largest amount of functional derangement." Not only are the usual symptoms of dyspepsia present, but hypochondriasis in its worst forms, sometimes verging upon temporary insanity, frequently adds to the already abundant suffering.

*Complication with Functional Cerebral Affection.*—This is sometimes very distressing. The pain may be general and moderate, or local, excruciating and unbearable. In some cases it is of an intermittent and neuralgic character, while, in others, it is so constant and overwhelming as to lead to the belief of its resulting from organic disease. Various convulsive affections, seemingly originating in the severity of the pain, frequently occur. Vertigo, morbid acuteness of the senses, various sympathetic affections of the digestive organs, are also often present. It is consoling to know, that the result of experience proves that these symptoms rarely have an organic origin. In this complication, thus contrasting it with some others, the sleep often continues good, the appetite remains in some degree, and emaciation is not very rapid.

*Complication with Affections of the Vascular System.*—The symptoms resulting from these are of less frequent occurrence, but very formidable in appearance, and excite the greatest anxiety in the minds of the friends of the patient. They are total loss of colour, œdema, palpitation, syncope, &c. Dr. Ashwell has found ascites rarely present, except in cases occurring at the more advanced periods of life, when it arises usually from some structural disease in the abdomen. Care must be taken not to attribute the icterode cast of countenance to liver disease, requiring the employment of mercury.

*Complication with Phthisis.*—This is usually the cause of death, when chlorosis terminates fatally. It is a question worthy of notice, whether chlorosis and amenorrhœa induce phthisis, or, whether these affections themselves do not rather arise from the originally phthisical tendency of the system delaying the approach of puberty, amid its other injurious influences. Although it is probable that chlorosis may in some cases produce phthisis, yet, in the majority of cases, it only developes the latent tendency to the disease. This opinion is confirmed by the fact, that however far the other complications of chlorosis may have extended, and however low they may have reduced the patient, they rarely pass into phthisis. In the present complication there may be an absence of the severe suffering found in some others, but the quick pulse, hurried respiration, rapid emaciation, and troublesome cough, too often replace these. Dr. Ashwell thinks the attention of the profession should be more directed than it has been to the too frequent occurrence of phthisis in this disease, especially as the fears of the patient and her friends are often lulled by the self-delusive nature of the disease. The prognosis is very dubious. Our hope of success consists in improving the condition of the blood, and our sign of having accomplished this is found in the encrease of flesh and in the diminished rapidity of the pulse, for, so long as this "beats 130, 120, or even 110 in the minute, it must not be supposed that any real amelioration has taken place." We must not be satisfied with the absence of auscultatory signs, but bear in mind, that the general condition of the patient is favorable to the deposition of tubercle. If we wait to adopt precautionary treatment until the stethoscope indicates the actual existence of the disease, it will be too late.

These various complications, which we have now considered, when they have long existed, render the case very confused, and difficult for accurate diagnosis. An obstinate leucorrhœa, moreover, is a frequent attendant, impairing the restorative powers, and materially retarding the cure. Simple chlorosis is usually a disease of early life; joined with amenorrhœa, it may be met with at any time between puberty and the cessation of the menses. The complication with phthisis is found chiefly between the period of puberty and the age of thirty.

### TREATMENT OF CHLOROSIS.

The treatment of simple chlorosis should be the type for the treatment of the other forms; but, a most grave error is too often committed, by considering it a local, not a constitutional disease; and ignorant practitioners, from the untimely use of drastics and emmenagogues, have yet farther reduced the already enfeebled powers, and facilitated the advent of pulmonary disease. The author deplores, in common with every medical man of proper feeling, the prevalent faulty notions on the subject of physical female education, leading as they do to the production of so many serious and fatal diseases: he believes that, were these amended, chlorosis would become a rare disease. Alas! many, many yet must be the victims sacrificed at the shrines of fashion and folly, ere mothers learn prudence, or fathers compel the observance of the dictates of common sense.

Our first attention must be directed to the improvement of the state of the digestive organs, for, how shall we amend the deteriorated condition of the blood, until the organs of nutrition are in a fitting state for its elimination. But here a prudent hand must guide the means: our object is not to excite excessive purging, as a direct mode of cure, but to secure the due relief of the bowels by aloes and rhubarb, sulphate of soda and manna, and, where alteratives are required, the hyd. c̄ creta. Mild cordials should be combined with the aperients. Warm clothing, regular exercise, and, when the state of the appetite will permit, meat diet and mild malt drink, are to be recommended. If we succeed in improving the state of the digestive organs, the general vigour is in some degree restored, and the complexion partially cleared, but the catamenia are seldom by this alone induced. Now is the appropriate period for the administration of *iron*, especially the *sulphate*, while, had this remedy been employed prior to the due regulation of the secretions of the alimentary canal, the symptoms would have become aggravated, and not relieved. Its effect, when given judiciously, is sometimes magical. In some cases the subcarbonate is better borne, and occasionally other tonics, as quinine, sarsaparilla, zinc, &c. effect the purpose.

As to *Emmenagogues*, they are best employed when the pallor has become diminished, the bowels more regular, and the blood both more abundant and of richer quality. *Iron* (and especially the iodide, when the strumous diathesis is associated with chlorosis) is often alone a sufficient emmenagogue. The use of the mustard hip-bath, and of the local salt shower-bath across the loins, are excellent adjuvants. The injection of the vagina with the strong ammonia (liq. ammon. ʒj. lactis ℔j.) has proved useful in the hospital. Dr. A. has great doubts of the utility of applying leeches and cataplasms to the mammæ; he has often seen electricity useful. Travelling, with the change of scene and of habits it necessitates, as also a visit to chalybeate waters, and a sea voyage, have often cured chlorosis. The treatment requires to be early adopted, and most perseveringly continued, perhaps for months. As the cure progresses the diet should be improved, and the patient permitted to take mild ale or porter, or, if these are disagreeable, a little negus with her meals.

*Treatment of the Complications.*—The *hæmatemesis*, which frequently occurs in these cases, is sometimes accompanied with so much pain and congestion in several organs, as to lead to the adoption of active means, such as bleeding, purging, lead, &c., but always, according to Dr. Ashwell's experience, with bad effect. Bleeding is seldom required, and active or long-continued purging is always injurious. The general symptoms of chlorosis will enable us to distinguish the local affection from one of an inflammatory origin, and lead us to adopt means for increasing the natural menstrual secretion, such as electricity, the mustard-bath, leeches to the vulva, moderate cupping of the loins, together with the use of emmenagogues and an occasional purge. In the *cerebral* affections local cupping, blisters to the nape of the neck, moderate and cordial aperients, change of air, cheerful occupation and society, with active out-of-doors pursuits, give most relief. In threatened *phthisis* an early and entire change of air may sometimes avert the impending mischief.

### II. AMENORRHŒA.

This Dr. Ashwell divides into Amenorrhœa of Retention, when at the proper age menstruation is absent, and Amenorrhœa of Suppression, in which the menses, having been once in existence, become suppressed.

### (A.) AMENORRHŒA OF RETENTION.

1. *From Deficiency, Malformation, or Structural Disease of the Organs of Generation.*—These cases are happily rare, and of course often incurable, as, for example, when the ovaries are absent or structurally diseased: menstruation may however occur when but a portion of an ovary remains. The absence of the ovaries confers a masculine appearance upon the female, and she is often submitted to most serious disorders of her health. In cases of absence of the uterus the general health has sometimes not seemed to suffer, which however is not the case when the menstrual fluid, although secreted, is prevented escaping by some malformation; and, if not liberated by a surgical operation, the accumulation may give rise to serious or even fatal symptoms.

*Treatment.*—This of course, where the organs are absent, is out of the question; but, where obstruction or malformation exists, judicious surgical interference will do much, although the risk of inducing peritoneal inflammation should be always borne in mind.

2. *From a slow and partial Development, or an entire Absence of Puberty.*—The period for the establishment of puberty varying in different individuals, the mere absence of menstruation at the usual epoch must not be regarded as disease; delicacy of constitution or idiosyncrasy may retard its development. Rare cases will sometimes occur, even independently of congenital malformations, in which puberty and menstruation are never established. If the amenorrhœa be very prolonged, the state of system characteristic of chlorosis will manifest itself. These cases usually terminate favourably, if violent means be abstained from, but months or even years may have to be spent in their treatment, which is of a similar description to that required for chlorosis.

3. *Amenorrhœa after Puberty.*—(a.) *As occurring in the Plethoric and Robust.*—This form of amenorrhœa, characterised by a state of congestion, or of active plethora, is usually found in the inhabitants of rural districts; it is generally curable, though often neglected. Fulness, and pain of the head, and pain in the lumbar region, with a full pulse, accompany this form, especially at the periods when menstruation should occur. Some women have naturally long intervals between their periods, plethora and dysmenorrhœa being at such times present. If the amenorrhœa be neglected or badly treated, it may become a most obstinate disease. It usually originates in a congested state of the uterus impeding its secretory powers. The symptoms of plethora may become subdued, and yet the case pass into one of ordinary chlorosis, the establishment of the catamenia being indefinitely delayed.

*Treatment.*—General bleeding is not required unless some important organ becomes congested. Cupping the loins or sacrum, and the application of leeches to the labia, thighs, or uterus, together with active purgatives, usually relieve sufficiently. The effectual use of the mustard hip or foot bath

(maintained at the temperature of 96° or 98° for an hour at a time), every or every other night, is very desirable, and active walking exercise should be insisted upon, but riding tends to encrease the uterine congestion. Dr. Ashwell has found small revulsive venesections (5 or 6 ozs.), about the period of the menstrual effort, very useful, but he cannot say the same for the practice of leeching the mammæ. Even when the plethoric state has become subdued, we should not be in too great haste to resort to emmenagogues, as some months may be required before the uterus will properly resume its functions. Where, however, true debility ensues, and chlorosis is threatened, they should be employed.

(b.) *As occurring in the Delicate, Hysterical, and Irritable Female.*—These cases are more common than those already noticed, the induction of menstruation being in them difficult and tedious, and, in spite of emmenagogues, they will often degenerate into cases of chlorosis complicated with amenorrhœa.

### (B.) AMENORRHŒA OF SUPPRESSION.

1. *Recent and Acute Suppression.*—This is especially brought about by mental emotion and cold. The symptoms vary according to the constitution of the subject. In the plethoric and healthy we may have congestion, or even inflammation of the uterus; in the delicate and nervous, spasmodic and neuralgic symptoms. Hysteria approaching to epilepsy, and partial paralysis, are sometimes present.

*Treatment.*—When inflammation exists (and it has been known even to pass into a state of gangrene), prompt and active bleeding are called for, the mere suppression being quite a secondary matter. In the delicate (in whom suppression is more apt to occur), the pain and other symptoms are rarely of an inflammatory origin, although the satisfactory determination of this point is sometimes very difficult. In these cases the pain is very fugacious and changes from place to place, often accompanied by hysteria and syncope. Here, even local bleeding is seldom useful, producing a metastasis, but not a removal of the pain. Active purging to relieve the bowels, usually overloaded, is desirable, while local baths and antispasmodics should also be had recourse to. Anodyne and antispasmodic clysters are sometimes almost magical in their effects; they should be small in bulk, and retained in the rectum for a considerable period, by pressing its orifice with a napkin. Sometimes, as the result of this treatment, the menses at once appear; but, on the other hand, when month after month passes without this being the case, a state of chronic suppression becomes established. After a first attack of suppression, every effort should be used, just prior to the next period, to induce the natural secretion. The warmth of the body should be carefully provided for, the bowels kept free, mental and physical excesses avoided, and the mustard baths used on alternate nights.

2. *Chronic Suppression.*—This may either result from the acute form, or come on gradually owing to constitutional delicacy or ill-health. It may also arise from organic disease of the organs of generation, or from a premature cessation of the menses. In considering these cases we must recollect that some women, although healthy, menstruate so slightly, that chronic suppression would seem otherwise to be at hand. Among the symptoms may be mentioned vertigo, obstinate headache, variations of temperature of the surface, loaded bowels, dyspnœa, palpitation, &c. If the disease continue unrelieved, the health may become broken up, and phthisis or organic abdominal disease ensue.

*Treatment.*—Many slight cases from cold, &c. will disappear without aid, and, where no effect is produced on the health, the case may be left to Nature. When abundant leucorrhœa is present, it should, owing to its debilitating effects, be checked as soon as possible. The cases occurring in nervous subjects are usually very protracted; and those yield much more easily, where some remains of congestion still exist, provided this be effectually relieved, before resorting to stimuli: when this is doubtful a careful examination of the uterus should be made. When debility exists, unaccompanied by plethora, stimuli and tonics are required.

### III. Emmenagogues.

The author does not believe that there are any medicines which exert a specific effect upon the menstrual secretion, but that there are several, which, by reason of their stimulating the uterus, become important auxiliaries. These means are contra-indicated in amenorrhœa and chlorosis, arising from malformation or absence of the generative organs, as also in cases of mere absence or slow development of puberty, or when the amenorrhœa is connected with phthisis or plethora. They are found useful in inactivity of the uterus, occurring after the establishment of puberty, where, neither plethora, or marked delicacy of constitution is present, as also in hysterical irritable women, in whose cases cordials and tonics have been tried in vain. In chronic suppression they are especially indicated. Their exhibition should be preceded by local depletion, regulated diet, and purgatives.

1. *Local Emmenagogues.*—The only powerful emmenagogue by which the uterus can be directly stimulated is *electricity.* Although employed with some success of late at Guy's, it is an uncertain remedy and should be very cautiously used. *Leeching the os uteri.*—If some leeches be applied by means of a glass, a few days prior to the period, and repeated several times, by removing the congestion, they will frequently reproduce the secretion. *Stimulant injections of the vagina.*—Dr. Ashwell speaks highly in favour of the ammoniacal injection. It should be commenced three days prior to the expected period, and should be retained in the vagina for ten or fifteen minutes, by closing the vulva with a napkin. It should produce a sense of heat, tingling, or even of pain, and should not be employed when congestion is present. He reprobates, as risking the excitement of peritonitis, the injection of the uterine cavity itself. *Mustard hip bath* is often very useful, but the patient should remain in it an hour each time. The exhibition of *mustard* by the mouth (gr. 8—12 ter quaterve ex M. camph), just prior to the menstrual period, is often attended with excellent effect. *Sexual intercourse* is sometimes a good emmenagogue, though of course, of but limited application. *Stimulating clysters* are much recommended by some authors, and Dr. Ashwell has used that of Dr. Schonlein (Aloes gr. x. mucil. ℥j. bis terve die)

with good effect. Leeching the vulva or pubes, stimulating the thighs, hypogastric or lumbar regions with embrocations, flesh-brushes, &c. &c. are all useful adjuvants.

2. *Constitutional Emmenagogues.*—The author thus expresses his opinion on *Mercury.*

"It is not to be used in slight cases, nor where there is extreme exhaustion, a predominant irritability, or a tendency to phthisical or strumous disease. But, in obstinate amenorrhœa, where other treatment has failed, where there is chronic inflammation or permanent congestion, and any evidence of incipient structural change, there is no remedy comparable to this...... As an alterative I have not used it successfully; but if salivation be produced and maintained, mercury often ensures decided and permanent benefit...... If the pulse becomes more rapid and less strong; if constitutional irritation and weakness daily increase; if there be cough or diarrhœa, these not having previously existed, the mercury should at once be discontinued. More frequently, in cases warranting its use, improved symptoms will follow moderate salivation........ The mercurial effect should be carried so far as to produce soreness of the gums and moderate salivation; and these should be kept up for twelve or sixteen weeks." 77.

*Iron.*—This excellent medicine is the more valuable, as, since it acts by improving the impoverished state of the blood, its effects are more likely to be permanent. Plethora and constipation must be removed before employing it, and it should be discontinued on the appearance of "giddiness, headache, sickness, and a quick or full pulse."

*Secale Cornutum.*—Dr. A. reports unfavourably of the emmenagogue powers of this medicine. It should be employed cautiously, as its long-continued use will be followed by irritation and spasm of the abdomen. It is most successful in relaxed and debilitated habits, and when determinate efforts are making by the uterus to establish its secretion. *Iodine,* by improving the condition of the blood in scrofulous subjects, is sometimes a very good medicine, but Dr. Ashwell has met with repeated failures. *Strychnine* he has used without success. *Nitre* occasionally exerts a beneficial effect, through the medium of the kidneys. *Aloes* should not be given in a congested state of the alimentary canal or uterus, but, when this is not present, it often determines the inactive uterus to secretion.

### IV. VICARIOUS MENSTRUATION.

This, when it neither deranges or exhausts the powers of the system, can hardly be called a disease: it usually occurs in the unmarried, (when married women suffer from it, they rarely conceive during its existence,) and as often in the weak as in the robust. The discharge, usually sanguineous, is occasionally leucorrhœal. "Some portion of the pulmonary and intestinal mucous tissues are thought to be the more common seats of the vicarious loss; but certain it is, that the nipples, the ears, the gums, the umbilicus, the axillæ, the bladder, any part of the skin, or mucous membranes, or the surface of an open ulcer, may occasionally by gush, more usually by slow transudation for several days, furnish the vicarious blood. In the regularity of its return, it seldom resembles the healthy function, although cases are recorded where the menstrual epoch has been exactly observed." The discharge is frequently preceded by local pain. It never terminates fatally; the uterus resuming its functions eventually, and the vicarious organ sustaining no injury.

*Treatment.*—If it occur often, and premonitory symptoms are present, emmenagogues, in the absence of plethora, may be used; while, if congestion of the uterus exists, the ordinary means for subduing it must be put in force. "A smart drastic purgative may not only prevent the vicarious attack, but also induce menstruation; and I have several times, after depletion, witnessed the good effects of electricity and the strong mustard bath, at a high temperature." If the hæmorrhage be great, it must be treated on general principles.

*Vicarious Leucorrhœa.*—In these cases "strictly speaking, there is amenorrhœa, because a mucous, instead of a sanguineous secretion, is furnished by the minute extremities of the uterine arteries. But there is activity instead of torpor; and it will be found, on inquiry, that all the symptoms denoting menstruation regularly appear, especially when this condition is vicarious of the catamenia at an early age." It is most common in delicate females, and in women exhausted from various causes. Dr. A. has known conception occur during this colourless menstruation. In early life, when it occurs only at intervals, the health is usually not much deranged, and, under favourable circumstances, natural menstruation eventually displaces it. The *treatment* is that required for vicarious menstruation, good air, tonics, and nutritious diet being often necessary to assist in establishing the natural functions.

### V. Dysmenorrhœa.

"Dysmenorrhœa is an important disease. It is very common, and produces extreme suffering—it often prevents conception, and, if pregnancy has occurred during its continuance, the woman is exposed to the risk of abortion. Although, in itself, it is not a fatal malady, yet it admits of proof, that malignant diseases have followed its protracted existence, and, lastly, it is exceedingly difficult to cure." 100.

Single women are most liable to it, and especially those of delicate, strumous, or phthisical constitutions. It is rare among those of a sanguine temperament. The mere painful state which precedes the menstrual secretion, though often excessive, does not constitute a case of dysmenorrhœa. The disease manifests itself under various forms.

1. *Irritable or Neuralgic Dysmenorrhœa.*—This occurs independently of inflammation or congestion. After an irregular condition of the menses occurring for some time, dreadful pain accompanies the emission, while the discharge itself is scanty and clotted. The degree and duration of suffering vary much in different cases, depending much upon the local organization of the parts, and the general susceptibility of the patient: it sometimes is horrible and protracted for days. The mammæ usually sympathize, but the constitutional disturbance is not great, and the strength, after the attack has subsided, remains little impaired. If, however, the case be neglected, and the disease established, the general health gives way, and complete anæmia and chlorosis may result. In the *plethoric* form the expulsive efforts made to discharge the clots are very violent; exactly resembling the throes of labour. The *congestive* form is often induced by emmenagogues improperly used, determining blood to the uterus. Here, too, intense sufferings are proved by the expulsion of small coagula, which are often mistaken for ova. Mackintosh, Capuron, and others, attribute the symptoms of dysmenorrhœa to *mechanical obstruction*, from a strictured state of the cervix, or a partial obliteration of the os uteri. Dr. Ashwell, without going so far as this, still believes, that in many cases such contraction exists, but alludes to some in which its dilatation was not followed by relief.

*Diagnosis*.—This is of importance in cases of suspected abortion. "The duration of the complaint, the nature of the menstrual secretion in former periods, the enlarged state of the uterus from congestion, as ascertained from examination by the vagina and rectum, independently of the physical characters of the product, are quite sufficient to satisfy any observer." The author quotes Dr. Montgomery's admirable observations on the distinction between true and spurious ova.

*Pathology*.—Much difference of opinion prevails upon this point; some consider the disease as always of a nervous, and others as of an inflammatory nature. The author believes that either of these states may prevail in different cases, for, while in some there is mere irritation, producing excessive suffering, in others, a low degree of inflammatory action exists, producing the false membrane, which is extruded with such difficulty; and which, if it continue long enough, may cause a degree of thickening of the os and cervix uteri sufficient to create a mechanical obstacle, and in other cases may even lay the foundation for organic disease.

*Prognosis*.—This disease is never directly fatal; and, if it be considered a mere neuralgia, however great the suffering, a favourable prognosis may be given. In patients predisposed to scirrhous disease, our opinion should be guarded. In the great majority of cases the affection is cured by medical treatment, child-bearing, or by the natural cessation of the menses.

"*Treatment*.—There are, in the treatment of every variety, two principal indications: to alleviate the urgent pain of the menstrual period, and to employ, during the intervals of the discharge, such remedies as shall restore to the uterus its healthy secretory power. Both are occasionally accomplished with difficulty; the first, however, is generally the most easy of fulfilment." 109.

In the usual or *neuralgic* form, the patient should employ the mustard hip-bath on the accession of the premonitory pains, repeating it three or four times in the twenty-four hours, and remaining in it half or three quarters of an hour, or even, if the pain is very violent, until faintness comes on, while nauseating diaphoretics should at the same time be given.

In mild cases the hip-bath and slight narcotics suffice. When the pain is very severe morphia and opium suppositories are required. When, from the forcing efforts made, there is reason to believe that a coagulum or false membrane is in process of expulsion, repeated doses of the *ergot* may facilitate it, and obtain at least an interval of ease. General bleeding is sometimes, local always, required: this latter is perhaps best performed by leeching the os uteri, or rather by scarifying that part, as recommended by Mr. Fenner.* Fomentations and anodyne injections of the vagina must not be neglected.

During the *intervals,* all the means calculated to restore the general health, as mild aperients and alteratives, chalybeates, fresh air, exercise, &c. must be resorted to. Dr. Ashwell has been disappointed in trying the volatile tincture of guaiacum, as recommended by Dewees. In the *congestive* and *plethoric* forms, occasional depletion is required, while the use of tonics requires great care. The author considers marriage as often remedial, although very bad cases do sometimes occur in married life. When *structural change* is dreaded, *mercury,* perseveringly employed, is indicated: it should be had recourse to when the disease is very obstinate, and the membrane habitually expelled; but, especially, when there is a thickened or indurated cervix. When the cervix is enlarged and hardened, the iodine ointment may be advantageously used.

### VI. Menorrhagia.

Under this head, Dr. Ashwell considers both cases of profuse menstruation, and cases of uterine hæmorrhage independent of pregnancy.

1. *Profuse Menstruation without Uterine Bleeding.*—Females somewhat advanced in life, and of a delicate habit of body, are more liable to this form than the young and robust. Climate and idiosyncrasy cause varieties in the quantities lost at the menstrual period, and our judgment must be formed principally from the effects upon the system. Menstruation may be excessive from the large quantity lost in a short time, or (more commonly) from a small discharge being continued over a very long period; or, again, from the return of the periods being too quick, " young and single women are more prone to the latter; while married females, weakened by childbirth, undue lactation and leucorrhœa, are obnoxious to the former variety." Leucorrhœa is usually present prior to or during the intervals. The symptoms are those which usually attend hæmorrhage. On examination per vaginam a flabby state of the various organs is found; the os uteri is somewhat patulous, but there is neither tenderness or induration. These patients are very prone to abortion and to prolapsus uteri. In a few cases plethora, but in most cases a delicacy of system, (which may be produced *inter alia* by repeated labors and abortions, undue lactation, excessive venery,) predispose to the disease. It is distinguished from the other forms of menorrhagia by the absence of coagula.

*Treatment.*—In the plethoric and robust the discharge is often salutary; and may be permitted to subside naturally. Moderate depletion (usually local) may be instituted prior to the menstrual period. In delicate and feeble women, stimuli and anodynes may be required; and, when the dis-

---

* See Medico-Chirurgical Review, No. 64, p. 620, April 1840.

charge is very great, the ergot is especially useful in checking it. The local application of, and injection into the vagina of cold water, and even the plug, (though rarely when the case has been well treated,) may be required. In the *interval*, means directed to the improvement of the general health, together with salt-water baths, vaginal injections, and cold sponging the loins and hypogastrium, must be put into requisition.

2. *Profuse Menstruation with Discharge of Blood.* (a.) *Acute or Active Menorrhagia.*—This is not very common, occurs chiefly in the robust, and is preceded by plethoric symptoms, which are indeed often relieved by the discharge. The frequent recurrence of the discharge eventually deranges the health seriously. The treatment is like that already named, and smart drastic purges are often of great utility. When spasmodic rather than inflammatory action is present, nauseating doses of ipecacuanha, and clysters of assafœtida and opium are the remedies. Great care is demanded in the management of the *interval*, and caution is especially required respecting tonics and stimuli, which, if injudiciously employed, only serve to keep up the discharge: thus Dr. Ashwell has known cases which obstinately resisted the ergot and tonics, cured by recurrence to antiphlogistics.

(b.) *Passive or Chronic Menorrhagia.*—This, occurring in the delicate, hysterical or exhausted female, is the most common form of the disease, and often arises from the neglect of early profuse menstruation, and from the injurious employment of stimuli. Every degree from the most trivial to the most dangerous prostration may occur, and, although the disease is in itself rarely fatal, yet it may give rise to dropsical effusions and other serious mischief.

*Treatment.*—The most absolute rest is requisite, and, in the aggravated cases, the same treatment which is required for puerperal hæmorrhages is required. Dr. Ashwell thus expresses himself on the subject of *astringent injections*.

"Astringent injections should rarely be used during the first few days of the menstrual period, as they often produce uterine spasm; but when coagula are passed, either alone or mixed with the catamenial fluid, the secretory function is either partially or entirely suspended, and injections may then be highly beneficial. It is essential that the patient lie down, when the injection is thrown into the vagina, that the pelvis be raised by placing a sofa cushion under the hips, so that the fluid may easily reach the upper extremity of the canal, and that whatever quantity be injected, it shall be retained for ten or fifteen minutes in direct apposition with the parts. To effect this, the nurse should make firm pressure on the vaginal orifice by a napkin accurately applied. Where these conditions are complied with, and where occasionally, in susceptible and irritable women, the injections are slightly warmed, so as to prevent the probability of the occurrence of uterine spasm and pain, I know practically that great good will generally result from their administration." 139.

Dr. A. employs the nitrate of silver, decoction of ergot, oak bark, acetate of lead, sulphate of iron, &c. &c. as materials of injections, and prefers the india-rubber bottles with ivory tubes to syringes. The following is our author's opinion of the *plug* in aggravated cases.

"During the flow, if alarming loss of blood seems to be approaching, the ergot and opium, injection of cold water and astringent lotions into the rectum, and, above all, plugging the vagina as far as the os, must be practised. Soft *dry* tow, slowly introduced in small quantities, till the passage is entirely filled, forms the best tampon or plug, and it may be allowed to remain unchanged for twenty-four or thirty hours. The patient will probably object to such a remedy and suffer slightly from its use; but neither of these circumstances are sufficient to justify the practitioner in giving it up. A silk handkerchief, lint, or linen, may be used, but they must be dry; if wet or saturated with moisture, their introduction is painful and difficult: dry, soft tow, in small pieces, is certainly far better. I am convinced that, in excessive menorrhagia, plugging is not sufficiently often resorted to." 140.

(c.) *Congestive Menorrhagia.*—This form usually occurs at the middle or more advanced periods of life. It possesses characters different from the other forms, and Dr. Ashwell is surprised that so little attention has been paid to it. It is very tedious, sometimes lasting for years, copious leucorrhœa often accompanying it. The author has not met with it prior to the ages of thirty-eight or forty, but he has met with modified attacks after menstruation might have seemed to have ceased. The effects vary according to the degree and continuance of the discharge, which sometimes continues for months without intermission, or degenerates into leucorrhœa. Prior to the repetition of a fresh irruption, there is often great bearing-down in the region of the uterus, and Dr. Churchill has observed great dysury frequently. The disease sometimes undergoes a natural cure by the occurrence of pregnancy, and more frequently from the cessation of the menses. There is often great difficulty in deciding whether organic disease is present, and this can only be ascertained by a careful examination per vaginam. Even when such disease does not exist we shall find " an encreased uterine bulk, fulness of the cervix and openness of the os uteri."

*Treatment.*—Where plethora is present, either general or local bleeding, and, if there be fulness or pain about the cervix uteri, scarification of that part, must be employed. Sexual intercourse and all kinds of stimuli must be avoided for some days prior to the menstrual periods. The various debilitating effects resulting from the excess of discharge, must be treated upon general principles. Astringent injections of the vagina are useful, especially when abundant leucorrhœa is present. Although ergot has often failed, and sometimes even produced ill effects, yet Dr. Ashwell speaks favorably of it. Lead and opium, turpentine, iron and opium, have in other cases been useful.

Dr. Ashwell considers menorrhagia may frequently be attributed to the avoidence of sexual intercourse, and the consequent congestion of the uterus and ovaries. " This abstinence is dangerously practised to avoid the risk of adding to the number of a family, already thought to be too numerous for the pecuniary means of its principal supporter." Loaded bowels and luxurious living are causes of menorrhagia, and purgatives used twenty-four hours prior to the expected attack are often very useful.

### VII. Leucorrhœa.

Of all the sexual diseases this is the most common: few mothers escape its attacks. The young and robust female is less liable to it than the delicate and older one. In its mild form it is so trifling a disease, that it is very often neglected by the patient.

"And yet I believe, if care were taken at this early stage, if ablution only was frequently practised, the tone of all the parts, and more particularly of the secretory membrane, would be regained, and further mischief entirely prevented: so far as my observation has gone; there is amongst female youth, and women generally, in this country, an unfounded dread of ablution of the external organs, either cold or tepid. The vicissitudes of our climate in some measure account for and justify the impression, but nevertheless it is too general, and extensively injurious." 159.

It may last for months or years, or sometimes during life; the ordinary secretion from the genital organs, is a glairy, lubricating, white-of-egg like fluid, but the leucorrhœal fluid varies according to the part whence it proceeds: thus, that from the vagina is more abundant and less viscid than the uterine, while, that proceeding from the interior of the cervix uteri, is more tenacious than either. It assumes every variety of appearance however, from that of the simple encrease of the natural mucus, to a state of muco-purulency, purulent ichorous, sanguineous, and varying-coloured discharge, especially in cases where inflammatory action or organic disease exists. An examination per vaginam, when inflammation is present, will discover a slight encrease in the size of the uterus and a tenderness of its cervix; at other times, this last is found soft and patulous: the speculum shews it to be sometimes red and at others pale.

Dr. Ashwell does not attempt to classify cases of leucorrhœa into uterine and vaginal: the diagnosis of the part of the mucous membrane affected is often difficult, and very frequently both portions are implicated. The vagina is however usually the seat of the disease, and leucorrhœa so originating is usually the least severe and most easily curable; when there is great constitutional disturbance, and the leucorrhœa is very obstinate, the uterus is usually the seat of the affection. The author describes four forms of the disease.

1. *Acute aud Mild Leucorrhœa.*—Cases of mild leucorrhœa, arising from excitement and congestion of the vessels of the secreting parts, are the most frequent, the locality of the discharge in such cases being usually situated in the glands at the entrance of the vagina. In some of these cases there is much local and constitutional irritation, especially if they be badly treated at the beginning, and, even under the best treatment, the case occasionally proves very obstinate and passes into the chronic form. Generally, however, leucorrhœa is a disease rather of weakness than of plethora. There is a class of cases well deserving of attention; they arise among women who indulge too freely in the pleasures of the table, and lead a sedentary life: corpulency, accompanied with debility, is produced; the abdominal and generative organs become loaded with blood, and their functions improperly performed. In these cases profuse menstruation and leucorrhœa are frequent symptoms, and require careful treatment, for, if the discharges be too suddenly arrested, apoplexy, or various serious abdominal diseases may ensue.

2. *Chronic and aggravated Leucorrhœa.*—This is the form of leucorrhœa which is so difficult of cure. It usually arises from early neglect or bad treatment. Yet, not unfrequently, a copious discharge of this kind seems to be habitual, proving a frequent cause of sterility (from the anæmia of the generative organs), and of prolapsus of the uterus and vagina, and eventually of much constitutional mischief. In young women, chlorosis and amenorrhœa, and even phthisis, may result. We must not, in cases of local suffering and fœtid leucorrhœal discharge, too hastily conclude that structural disease of the uterus exists, but must resort to careful manual and ocular examination. With some women, and especially married women of a leuco-phlegmatic habit, whose constitution has been weakened by sexual excess, menorrhagia, abortion, or other debilitating cause, a constant leucorrhœal drain is sometimes kept up, producing few marked symptoms, while nevertheless the appearance of the patient is that of one suffering from a debilitating disease.

3. *Symptomatic Leucorrhœa.*—With many symptoms in common with ordinary leucorrhœa, there are several special ones, varying with the peculiar causes of the disease, and requiring a special treatment. Examinations carefully made, and frequently repeated, can alone assure us whether severe cases depend upon organic disease or not.

4. Dr. Ashwell makes some interesting remarks upon a *peculiar form of leucorrhœa*, which in its nature somewhat resembles hydrometra. The patient, after having long suffered from muco-purulent leucorrhœa, finds the discharge cease; a sense of fulness of the uterus and neighbouring organs follows, and, after it has endured during a varying period, from four to six ounces of a fluid, exactly resembling pus, comes away in a gush, and is eventually followed by a re-establishment of the muco-purulent secretion. If an examination per vaginam be made just prior to the sudden discharge, the uterus is usually found somewhat enlarged, the cervix swollen and tender, and the os closed partially. The cases are rare and usually difficult of cure. "I have never seen the affection in young females. Married women, and particularly widows, or those in whom the reproductive organs having been employed are so no longer, seem to be its frequent subjects."

*Causes of Leucorrhœa.*—As already mentioned, leucorrhœa occurs especially in the delicate and strumous, from causes inducing encreased action or inflammation of the various surfaces. These are especially cold, moisture, and excitement followed by debility, arising from many causes, as sexual excess, abortion, menorrhagia, lactation, &c. The irritation of a pessary or of stimulating injections has produced the disease; other causes of irritation act *indirectly*, and by these the chronic form of the disease is produced. Thus we may see it result from amenorrhœa, as also from irritation of the digestive organs, or of the spinal marrow.

*Pathology.*—Leucorrhœa arises from two different conditions, viz. hyperæmia, or encreased action of the secretory vessels, and debility, whether original, or resulting from the continuance of the former state.

"By some authors nearly all the cases are supposed to depend upon weakness, excepting such only as are accompanied by symptoms of inflammatory action. There is truth in this opinion, if the examples be included where the leucorrhœa, having been of the first kind originally, has, by its continuance, terminated in the opposite state. Let it however be remembered, that it does not necessarily follow because the system generally is delicate, that the vagina and uterus must of necessity be in a state of anæmia. Still, original or acquired feebleness of system may give encreased efficacy to the various exciting causes of this prevalent malady." 171.

*Diagnosis from Gonorrhœa.*—This is very difficult and sometimes quite impossible, seeing that leucorrhœa is itself often decidedly purulent, and may produce a discharge from the male organs, the disease so produced being however very mild, easily cured, and seldom followed by gleet.

"The perplexity, therefore, of these cases, is fully admitted; and it will often happen, that where we are most anxious to arrive at a positive conclusion, we shall be least able to do so. At all events, it behoves the practitioner to be extremely tenacious of the reputation and happiness of parties thus circumstanced. It is always his duty to cure the disease, but rarely to venture upon the exposition of its nature. If he can positively affirm that it is of simple origin, let him do so, if suspicion has been aroused; if not, it is better to avoid any distinct allusion to the matter." 175.

*Treatment.*—In *simple* cases, wherein the natural discharge from the parts is merely encreased in quantity, rest, abstinence from local and general stimuli, aperients, and a mild astringent wash, will usually shortly effect a cure. In the *inflammatory* form, which is rarer, local or general bleeding should be employed, and if the cervix uteri is swollen, red and tender, it should be leeched or *scarified*. "I have now several times scarified (not punctured) the neck of the uterus by a common lancet, mounted on a piece of whalebone, with marked benefit. The pain of the incision is most trifling; there is no ulceration or suffering afterwards, and in 24 hours the cervix generally seems to be entirely free from congestion." In one case he mentions, four or five ounces of blood were thus procured in a quarter of an hour. Local baths, aperients, salines and narcotics, are all to be employed. Astringents must be delayed until the inflammatory action has been subdued and the discharge becomes more abundant, or they will do harm. At the proper time these injections, together with tonics and stomachics, are very useful. In favorable cases a cure soon results from these means, but in other cases an *obstinate oozing* of discharge continues, producing from its prolonged continuance effects, which never would have been feared from its quantity. These cases much resemble the drainings of passive menorrhagia, and like them are often cured by the injection of three or four ounces of tepid (and after a while cold) fluid into the rectum night and morning. When the leucorrhœa continues obstinately and in large quantities, an examination per vaginam should be instituted, in order to distinguish those cases which arise from organic or specific disease, from those which result from what Hunter called the "habit of action:" and this is especially requisite when the discharge is fetid or acrid. In these cases a great variety and constant change of astringents will be required, and should be used in conjunction with tonics and other means for the establishment of the health. The nitrate of silver forms the best material for injection in obstinate cases. Stimuli, such as turpentine, lytta, and copaiba, are often given with advantage in these chronic cases.

An inveterate leucorrhœa cannot always be safely cured, without encreasing other excretions and observing a spare diet, especially when it has succeeded to suppressed or diminished discharges of any kind; while in women of full habit of body and luxurious modes of life, a seton may even be required. Our attention must be especially directed to this point in patients of a somewhat advanced age, in whom congestions of various organs are more probable, as also in the strumous and enfeebled subjects. "After the cure of habitual leucorrhœa, ablutions of cold water, at least, if not injections into the vagina, should be daily practised; avoiding their use for a few days before and subsequent to menstruation." Dr. Ashwell, alluding to the success said to attend the practice of the French physicians, of injecting the cavity of the uterus, justly represents it as fraught with danger.

### VIII. Inflammation of the Cervix Uteri.

This disease, first accurately described by Sir C. Clarke, Dr. Ashwell considers of infrequent occurrence, having seen but 20 examples out of 1000 cases of sexual disease at Guy's. It rarely occurs in single women, and is most common between the age of 20 and the period of cessation of the menses, and it has been observed to occur soon after marriage. The characteristic symptoms are an opaque white discharge, and distressing pain in the region of the pubes and sacrum, "aggravated by any circumstance which causes pressure centrally in the pelvis:" there is pain also during intercourse. The constitutional symptoms are slight, and frequently the menstruation is not deranged. When neglected the affection degenerates into inveterate leucorrhœa, and may lead to excessive anæmia. The *diagnosis* of the disease is derived from discovering pain of the cervix on pressure, and the opaque nature of the discharge, which is quite white or grey, and easily miscible (its test) with water. "Let it however be remembered, that this creamy discharge is rarely copious and free from admixture, except in rising from bed in the morning, the time which ought to be chosen for examination." *Treatment.*—In severe cases local bleeding is required, while in those of a slighter character, baths and tepid anodyne injections suffice: the poppy hip-bath used for an hour twice a day is especially useful. Aperients should be given, and sometimes anodynes or suppositories are needed, to allay the irritation of the bladder, which also occasionally requires the use of the catheter.

### IX. Disorders attendant on the Decline of Menstruation.

Dr. Ashwell considers that the opinion is too generally entertained, that the decline of this function must necessarily be attended with illness: doubtless, owing to the faulty state of the physical education of females, and to the evil influences they are exposed to in modern society, the ill effects at this epoch are frequent enough. In some cases, however, the health becomes improved and not deteriorated. The age at which the cessation occurs varies: generally it does so between 47 and 50, sometimes at 45, or in a few cases as early as 30 or between 30 and 40. In some, the change is completed in a few months, while in others, years are occupied. The author arranges the morbid affections which result in the order of their frequency.

1. *Functional Derangements of the Brain and Nervous System.*—These are the most frequent results; indeed, hysterical and nervous symptoms so frequently accompany this change, as to call for little attention; they however sometimes reach such a height as to verge on temporary insanity. Soothing measures are found best adapted to these cases, and those of an irritating character should especially be avoided.

2. *Increased Action and Congestion of various Organs* are next in frequency, and are far from being rare, especially in the plethoric and free livers: a tendency to fulness often continues long after the cessation is complete, several persons being then liable to hæmorrhagic diseases of the head and chest. Pain and various symptoms referable to a plethoric state of the vessels of the brain are here present, the diseases of the skin are very obstinate under these circumstances, and indeed any organ (especially the uterine) may be suffering from congestion or inflammation. In *treating* these affections we should be careful not to fall into the prevalent error of regarding them as the results of debility rather than plethora, for remedies exhibited under this idea not only may encrease the state of congestion of important organs, but may also lay the foundation for structural changes. Still we must be cautious in the use of antiphlogistics, for, if these be carried too far, anæmia will result. Thus a medium practice is the best: if there is plethora, small bleedings, aperients, and abstinence will be required. Issues and setons, formerly so frequently employed, are now rarely used. Local baths and friction are useful. Care must be taken not to confound the state of pregnancy with the cessation of the menses, or *vice versâ*—errors easily made under some circumstances.

3. *Lesions of Structure and Malignant Disease.*—Dr. Ashwell doubts the propriety of the opinion, which connects the period of the cessation of the menses with the original production of organic disease of the breast and uterus; believing, however, that a latent tendency may then become developed, especially when the uterus is in a state of congestion, and means for its relief have been neglected. Frequent examinations of the suspected organs are essential to the formation of a satisfactory opinion.

---

In concluding our analysis of this work, we may again express our opinion that it is an excellent one, and calculated to be of great use to the young practitioner, especially as it is enriched (and not overburdened, as is too often the case) with a selection of illustrative cases, and a very useful collection of prescriptions. One of Dr. Ashwell's reasons for its publication is admirable. "I have long entertained the opinion, that practitioners who hold important public appointments are bound, so far as their sources of authentic information can be made subservient, to improve and encrease the common stock of professional knowledge." Would that more of the medical officers of large institutions would follow his example!

